# Revolutionizing Cytology and Cytopathology with Natural Language Processing and Chatbot Technologies: A Narrative Review on Current Trends and Future Directions

**DOI:** 10.3390/bioengineering11111134

**Published:** 2024-11-11

**Authors:** Andrea Lastrucci, Enrico Giarnieri, Elisabetta Carico, Daniele Giansanti

**Affiliations:** 1 Department of Allied Health Professions, Azienda Ospedaliero-Universitaria Careggi, 50134 Florence, Italy; andrea.lastrucci@unifi.it; 2 Facoltà di Medicina e Psicologia, Sede Ospedale S. Andrea via di Grottarossa 1035, Università Sapienza, 00189 Roma, Italy; enrico.giarnieri@uniroma1.it (E.G.); elisabetta.carico@uniroma1.it (E.C.); 3 Centro TISP, Istituto Superiore di Sanità, via Regina Elena 299, 00161 Rome, Italy

**Keywords:** cytology, cytopathology, natural language processing, chatbot, ChatGPT

## Abstract

The application of chatbots and Natural Language Processing (NLP) in cytology and cytopathology is an emerging field, which is currently characterized by a limited but growing body of research. Here, a narrative review has been proposed utilizing a standardized checklist and quality control procedure for including scientific papers. This narrative review explores the early developments and potential future impact of these technologies in medical diagnostics. The current literature, comprising 11 studies (after excluding comments, letters, and editorials) suggests that chatbots and NLP offer significant opportunities to enhance diagnostic accuracy, streamline clinical workflows, and improve patient engagement. By automating the extraction and classification of medical information, these technologies can reduce human error and increase precision. They also promise to make patient information more accessible and facilitate complex decision-making processes, thereby fostering greater patient involvement in healthcare. Despite these promising prospects, several challenges need to be addressed for the full potential of these technologies to be realized. These include the need for data standardization, mitigation of biases in Artificial Intelligence (AI) systems, and comprehensive clinical validation. Furthermore, ethical, privacy, and legal considerations must be navigated carefully to ensure responsible AI deployment. Compared to the more established fields of histology, histopathology, and especially radiology, the integration of digital tools in cytology and cytopathology is still in its infancy. Building on the advancements in related fields, especially radiology’s experience with digital integration, where these technologies already offer promising solutions in mentoring, second opinions, and education, we can leverage this knowledge to further develop chatbots and natural language processing in cytology and cytopathology. Overall, this review underscores the transformative potential of these technologies while outlining the critical areas for future research and development.

## 1. Introduction

### 1.1. The Impact of Digitalization on Cytology and Cytopathology: Transforming Diagnostic Practices

Digitalization in cytology and cytopathology represents a significant technological innovation that involves using digital tools to capture, store, analyze, and share cellular images and data [1]. This process offers numerous benefits [2,3]. Firstly, it enhances diagnostic accuracy by providing high-resolution images, enabling more detailed analysis of cells and their abnormalities. The integration of artificial intelligence tools can support pathologists in interpreting images by automatically highlighting suspicious areas, contributing to more accurate and timely diagnoses [4].

Furthermore, digitalization allows for efficient preservation and archiving of digital images, which can be easily accessed for future consultations, comparisons, and patient follow-up [2,3]. This aspect is particularly advantageous for telepathology, where pathologists can examine samples and provide consultations remotely, making diagnosis possible even in remote regions or in the absence of local specialists.

Automation is another significant benefit offered by digitalization. Advanced systems can automate various processes, from slide scanning to generating diagnostic reports, improving operational efficiency and reducing the time required to obtain results [1,5].

However, the transition to a digitalized system is not without challenges. Implementing these technologies requires significant investments in equipment and infrastructure, as well as training personnel to ensure the effective use of new tools [6]. Additionally, data security and patient privacy protection are critical issues that must be carefully addressed.

The digitalization in cytology and cytopathology offers a range of advantages that can significantly improve the quality of care and efficiency of diagnostic processes [2,3].

In the initial phase of digital cytology, high-resolution images of cellular samples are captured using advanced imaging techniques, such as digital pathology scanners, which enable detailed visualization of cell morphology. The image is subsequently transformed into a virtual representation known as a digital slide. In the following phase, trained cytologists evaluate these digital slides using specialized software that functions as virtual microscopes, allowing them to analyze cellular characteristics and patterns to identify abnormalities indicative of various diseases.

However, the success of this transition depends on overcoming the associated technical, economic, and organizational challenges [6].

Digital cytology and cytopathology, together with digital histology and histopathology, are part of the broader field of digital pathology [7,8]. Unlike digital radiology, which advanced more rapidly, digital pathology has experienced a slower adoption rate, primarily due to the delayed integration of the DICOM (Digital Imaging and Communications in Medicine) standard, specifically in the form of DICOM WSI (Whole Slide Imaging) [9,10]. This slower integration process has also impacted the implementation of new Artificial Intelligence (AI)-based technologies, which depend heavily on robust digital frameworks [9].

Digital pathology, encompassing both cytology and histology, involves the digitization of slides and specimens, enabling storage, analysis, and sharing of high-resolution digital images [7]. This integration allows pathologists to work remotely and utilize advanced AI algorithms for more accurate and efficient diagnoses. The challenges in integrating DICOM WSI, necessary for standardizing whole slide imaging, have been a significant factor in the slower progress compared to digital radiology. These challenges include managing larger file sizes and ensuring precise image rendering, which are critical for accurate analysis and diagnosis.

The adoption of AI technologies in digital pathology, such as algorithms that identify patterns and anomalies in digital slides, hinges on a stable digital infrastructure. The initial delays in integrating DICOM WSI have consequently slowed the deployment of these AI tools, as they require standardized data formats and efficient image storage and sharing capabilities. The successful integration of these technologies is essential for leveraging AI’s full potential in enhancing diagnostic precision and optimizing workflow processes in pathology. Overall, digital cytology and histology are not separate from digital pathology but are integral components that, together with robust digital frameworks and AI technologies, form a comprehensive and evolving field. The unified approach to digital pathology, including the adoption of DICOM WSI [11] and AI [12], is crucial for advancing medical diagnostics and patient care.

### 1.2. Integrating Chatbots and NLP with Digital Imaging

Integrating chatbots and NLP with digital imaging is revolutionizing the healthcare domain, enhancing both diagnostic practices and patient interactions. In the realm of digital imaging, chatbots and NLP technologies offer powerful tools for improving the efficiency and accuracy of medical diagnostics [13,14].

While both chatbots and Natural Language Processing (NLP) are involved in enabling machines to interact with human language, they serve distinct roles and operate at different levels of complexity [13,14].

Chatbots are applications designed specifically to simulate conversation with users, often through text or voice interfaces. Their primary purpose is to engage in dialogue, answer questions, or assist users in performing tasks, such as booking an appointment, troubleshooting a problem, or providing customer service. Chatbots are typically user-facing and focused on interaction. In the past, many chatbots were rule-based, following pre-defined scripts and responding based on a set of rules or patterns. However, today, when we refer to chatbots, we typically mean AI-driven chatbots that use advanced technologies like machine learning and NLP to generate more flexible, natural, and dynamic responses. These AI chatbots can understand context, handle more complex interactions, and improve over time through learning.

NLP, by contrast, is a much broader field within artificial intelligence that focuses on the ability of computers to understand, interpret, and generate human language. While chatbots often rely on NLP to process and understand user inputs, NLP itself encompasses a wide array of tasks and applications beyond simple conversation. For instance, NLP is used in sentiment analysis, where machines analyze text to determine the emotional tone, in machine translation to convert text from one language to another, and in text summarization to condense large amounts of information into shorter summaries. NLP technologies are also used in tasks such as speech recognition (converting spoken words into text) and information retrieval (extracting relevant data from large text corpora).

In essence, chatbots are one application of NLP but not synonymous with it. They use NLP as a tool to better understand language and generate meaningful responses, but NLP extends far beyond just powering chatbots. While chatbots are specifically designed for conversational purposes, NLP encompasses a broader set of language-related capabilities that are applied in various fields, such as translation, document analysis, and automated content generation.

In short, chatbots—particularly AI-driven chatbots—are conversation-focused tools that often use NLP to understand and respond to human language, whereas NLP is the underlying technology that deals with the complexities of processing and interpreting natural language across many different use cases.

Chatbots and NLP [13], powered by AI, can assist healthcare professionals by providing immediate access to patient information, medical records, and diagnostic data. These tools facilitate quicker decision-making and streamline workflows, enabling clinicians to focus more on patient care. For instance, a chatbot can help sort and retrieve relevant imaging data or assist in preliminary diagnostic assessments by processing textual data associated with medical images.

NLP [14], on the other hand, enhances the interpretation of imaging reports and medical notes by extracting meaningful information from unstructured text. This capability allows for better integration of imaging findings with clinical narratives, improving the coherence and comprehensiveness of diagnostic reports. NLP can also be used to automate the extraction of critical information from large volumes of text, such as radiology reports or patient histories, making it easier for healthcare providers to access and analyze relevant data.

Together, chatbots and NLP technologies can facilitate more effective communication between patients and healthcare providers by automating appointment scheduling, answering routine questions, and providing follow-up information. This integration not only improves patient engagement and satisfaction but also helps reduce the administrative burden on healthcare staff.

### 1.3. Integrating Chatbots and NLP with Digital Cytology and Cytopathology: Transforming Diagnostic Efficiency and Patient Interaction

Integrating chatbots and NLP [13,15,16,17,18] with digital cytology and cytopathology could present significant potential benefits and therefore the following key questions to explore:

- Diagnostic efficiency: Chatbots could automate administrative tasks, while NLP might quickly analyze and interpret text from cytological reports, enhancing diagnostic speed and accuracy;
- Data integration: NLP could correlate textual descriptions with digital images, providing a more comprehensive view of patient data and aiding pattern detection;
- Patient interaction: Chatbots could handle patient inquiries and provide information about tests and results, improving communication and satisfaction. NLP could help tailor follow-up care based on patient feedback;
- Remote collaboration: Chatbots might facilitate remote consultations, and NLP could streamline the presentation of findings, enhancing collaboration among pathologists and specialists;
- Report generation: NLP could assist in drafting diagnostic reports, speeding up documentation, and ensuring consistency.

Overall, these technologies have the potential to significantly improve diagnostic processes, patient support, and collaborative efforts in digital cytology and cytopathology, though practical implementation and further development are needed.

### 1.4. Purpose of the Study

We aimed to conduct a review in this field

The purpose of conducting a focused review on the integration of NLP and chatbots with digital cytology and cytopathology is to explore and understand the transformative potential these technologies hold for the field. As digital cytology and cytopathology increasingly become central to diagnostic practices, integrating advanced technologies such as NLP and chatbots could enhance diagnostic accuracy, streamline workflows, and improve patient interactions.

The purpose of this narrative review is to examine the role of the NLP and chatbots in cytology and cytopathology, focusing on its contributions, opportunities, challenges, and recommendations for ethical AI development.

Specific aims:

1. Evaluate contributions: categorize experiences in the field, highlighting the emerging themes that illustrate its impact;
2. Explore opportunities and challenges: identify the opportunities as well as the key challenges that still need to be addressed.

## 2. Methods

A narrative review of reviews was conducted, focusing on the field of the intersection of cytology/cytopathology with NLP and chatbot. A standardized checklist for narrative reviews, the ANDJ Narrative Checklist available online [19], was used. The ANDJ checklist with a brief description is reported in the Supplementary Materials. The search was based on targeted searches on Google Scholar, PubMed, and Scopus. 

The composite key was “*((chatbot[Title/Abstract]) OR (NLP[Title/Abstract]) OR (chatgpt[Title/Abstract]) OR(natural language processing[Title/Abstract]) OR (natural language model[Title/Abstract])) AND ((cytopathology [Title/Abstract]) OR (cytology [Title/Abstract]))*”.

The selection of the component elements for this overview was carried out based on five parameters (N1–N5), as in [20], which were evaluated on a scale from 1 = minimum to 5 = maximum, and one parameter (N6) with a binary assessment (Yes/No). These parameters are as follows:

N1. Is the rationale for the study clearly stated in the introduction?

N2. Is the design of the work appropriate?

N3. Are the methods clearly described?

N4. Are the results clearly presented?

N5. Are the conclusions justified and based on the results?

N6. Did the authors disclose all conflicts of interest?

All selected studies needed to meet the parameter N6 with a “Yes” and have parameters N1–N5 scored above 3.

Further keywords were also used to select references in the introduction and for the discussion where other imaging domains were also investigated for comparisons: *NLP, chatbots in healthcare, cytology diagnostics, cytopathology analysis, histology AI applications, histopathology automation, radiology AI integration, digital pathology, AI in medical diagnostics, medical imaging analysis, diagnostic automation, clinical workflow optimization, patient engagement technology, medical data standardization, AI bias and fairness, healthcare data integration, AI ethical considerations, machine learning in pathology, automated medical reporting, and healthcare chatbot applications.*

## 3. Results

In total, 11 studies were selected [21,22,23,24,25,26,27,28,29,30,31], comprising all the relevant research identified on PubMed, excluding editorials, letters, and comments.

The results were systematically organized into multiple subsections to provide a comprehensive understanding of the findings.

Section 3.1 presents trends observed across the studies, enhanced by graphical representations that illustrate the data visually. This subsection aims to highlight overarching patterns and significant shifts within the field, offering a clear view of how the research landscape has evolved over time.

Section 3.2 addresses Specific Aim 1, which is to evaluate contributions by categorizing experiences in the field and highlighting the emerging themes that illustrate their impacts.

Section 3.3 addresses Specific Aim 2, which is to explore opportunities and challenges by identifying the opportunities as well as the key challenges that still need to be addressed.

An analytical excerpt that provides a deeper examination of the results is reported in the Supplementary Materials.

### 3.1. The Trends in the Studies on NLP and Chatbot in the Field of Cytology or Cytopathology

A search was conducted on the PubMed database using the search criteria outlined in the first search key reported in Box 1. This search yielded a total of 15 studies on the use of NLP in the field of cytology or cytopathology.

Box 1 **The proposed composite keys.**

*((chatbot[Title/Abstract]) OR (NLP[Title/Abstract]) OR (chatgpt[Title/Abstract]) OR(natural language processing[Title/Abstract]) OR (natural language model[Title/Abstract])) AND ((cytopathology [Title/Abstract]) OR (cytology [Title/Abstract]))*

*((chatbot[Title/Abstract]) OR (NLP[Title/Abstract]) OR (chatgpt[Title/Abstract]) OR(natural language processing[Title/Abstract]) OR (natural language model[Title/Abstract])) AND ((histology[Title/Abstract]) OR (histopathology [Title/Abstract]))*

Of the 15 articles retrieved, only 1 is a review, 1 is a comment, 1 a letter, and 2 are editorials. The oldest article in this collection was published in 2012. This theme has been recently investigated by any authors and the Figure S1 (see Supplementary Materials) reports the temporal trend of studies retrieved based on the in the first search key given in Box 1.

As shown in Figure S1, research on this topic has grown recently, with nearly all the articles (*n* = 13, 86.6%) being published in the last five years. This recent growth is due to the increasing use and dissemination of NLP programs in the medical field. Despite the recent increase in published articles on this topic, a comparison with the second key search reported in Box 1 reveals that the application and use of NLP is more extensively discussed and debated in the field of histology and histopathology rather than in cytology and cytopathology. The temporal trends of published articles for each key search are illustrated in Figure S2 (see Supplementary Materials).

Figure S2 shows some overlap between the temporal trends in the publication of articles on the use of NLP in cytology and cytopathology. For both trends, the peak in the number of articles published on these topics has occurred in the last five years. Although the temporal trends are similar, the number of published articles on the integration of NLP in histology and histopathology is significantly higher than in cytology and cytopathology. In addition, Figure S2 shows that, in contrast to the trend in cytology and cytopathology, there was already an increase in articles on the use of NLP in histology in the period 2014–2019. Another notable difference between the two fields analyzed is the number of published reviews and systematic reviews on the integration of NLP (*n* = 5) in the fields of histology and histopathology. This indicates that the topic is highly debated, and authors often summarize the existing evidence in the literature. In contrast, there are few reviews or systematic reviews on the use of NLP in cytology and cytopathology.

Despite the limited number of studies published on the integration of NLP in cytology and cytopathology, the recent peak and increase in articles published on this topic suggests the need for a review that summarizes and analyzes the evidence available in the literature. Such a review would provide readers with a clear and detailed framework and the current state-of-the-art methods regarding the use and application of NLP in this medical field. Given the recent implementation and widespread availability of NLP software, a review can also serve as an excellent tool to analyze the use of specific software and the results obtained from their application in the fields of cytology and cytopathology. This would provide the reader with a comprehensive overview of the available software and their respective findings in these medical fields.

### 3.2. AI Applications in Cytology and Cytopathology: Mapping and Categorizing Chatbot and NLP Contributions

The key elements of the studies have been synthesized into two tables. The first table (Table 1) provides a specific categorization for each study. The second table (Table 2) presents emerging themes identified through a comparative analysis of the studies, highlighting their intersections.

This dual approach allows us to appreciate both the specific contributions of each study and the overarching trends and connections that span across multiple research efforts. By examining the fine details and macro areas of focus, we gain a comprehensive understanding of how various studies intersect and complement each other, revealing a more nuanced and interconnected landscape of research in this field

Starting with the fine categorization, Table 1 summarizes the results in this direction of the overview.

Table 2 emphasizes the extensive range of applications for chatbot and NLP technologies in cytopathology and medical diagnostics. By delineating the broad thematic areas identified across various studies, this table offers a comprehensive comparative analysis that illustrates how these advanced technologies are transforming the landscape of medical diagnostics. It highlights the differences and similarities in their implementation and impact, showcasing the effectiveness of AI-driven tools across diverse diagnostic contexts—from enhancing diagnostic accuracy to streamlining workflow processes. This comparison not only underscores the versatility of these technologies but also reveals critical insights into their roles in advancing medical practice.

The following are the broad areas identified through the included the studies:

1. AI and NLP in cancer diagnosis

The development of AI and NLP technologies has enhanced the precision and efficiency of cancer diagnosis. Studies like Mese et al. [21] and Malik and Zaheer [22] have explored AI-driven models for diagnosing thyroid nodules and assisting in pathological cancer diagnosis, respectively. Mese et al. demonstrated how a deep learning model, aided by ChatGPT, can accurately analyze thyroid nodules using ultrasound images, highlighting the potential of AI in supporting clinical decisions. Malik and Zaheer discussed the use of ChatGPT for integrating complex pathology workflows, emphasizing AI’s role in refining cancer diagnostics.

In addition to these studies, Rozova et al. [25] and Nandish et al. [27] have explored AI and NLP applications in detecting invasive fungal infections and classifying breast lesions. Rozova et al. used NLP to analyze cytology and histopathology reports for identifying fungal infections, while Nandish et al. developed a multilevel classification approach for breast lesions, demonstrating AI’s utility in diverse diagnostic contexts.

2. NLP for pathology and cytopathology

NLP has proven to be a powerful tool in the field of pathology and cytopathology. Giarnieri and Scardapane [23] reviewed advancements in machine learning and neural networks for pathology, noting the improved accuracy in detecting and classifying various pathologies. This research underscores the growing role of AI in processing and interpreting large datasets in pathology.

Similarly, Hsu et al. [26] and Nandish et al. [27] focused on applying NLP techniques to pathology reports. Hsu et al. used FastText™ to classify cervical biopsy diagnoses with high accuracy, while Nandish et al. applied NLP to automate the classification of breast lesions from cytopathology reports. Both studies highlight the potential of NLP to reduce manual effort and improve diagnostic accuracy in pathology.

3. AI-enhanced screening and decision support

AI’s role in screening and decision support has been transformative, particularly in enhancing participation and optimizing clinical decisions. Selmouni et al. [28] investigated the use of a chatbot-based decision aid to improve cervical cancer screening participation among disadvantaged women. Their study illustrates how AI tools can bridge gaps in healthcare access and increase engagement in preventive measures.

Wagholikar et al. [30] and Nguyen et al. [31] developed systems to automate cervical cancer screening recommendations and classify pathology reports for cancer registry notifications. Wagholikar’s decision support system integrates national guidelines to assist clinicians, while Nguyen’s system focuses on automating the classification of cancer-notifiable reports. Both studies demonstrate AI’s potential to streamline clinical workflows and ensure adherence to best practices.

4. Data extraction and classification with NLP

The automation of data extraction and classification through NLP has become a critical area of focus. Oliveira et al. [29] developed an NLP algorithm for identifying HPV-associated cancers and precancers, showcasing how NLP can facilitate efficient surveillance and research. Similarly, Nguyen et al. [31] designed a system for classifying pathology reports to streamline cancer registry notifications.

The application of NLP to extract and classify data from unstructured clinical text offers substantial benefits, including improved accuracy and reduced manual processing. The studies reviewed highlight how NLP can transform traditional data handling practices, making it possible to handle large volumes of medical data with greater efficiency.

Overall, Table 2 highlights that the integration of AI and NLP/chatbot into this field of medical diagnostics represents a significant advancement in healthcare technology. From enhancing cancer diagnosis and pathology workflows to improving screening participation and automating data classification, these technologies are reshaping how medical information is processed and utilized. The studies discussed demonstrate the diverse applications and potential benefits of AI and NLP, highlighting their role in improving diagnostic accuracy, optimizing clinical decision-making, and advancing public health initiatives. As these technologies continue to evolve, their impact on medical diagnostics is likely to expand, offering new opportunities for enhancing patient care and healthcare delivery.

### 3.3. Opportunities and Challenges in Implementing Chatbots and NLP for Cytology and Cytopathology

A comparison among the studies reveals opportunities and areas needing improvement. This analysis highlights both the potential benefits of integrating chatbots and NLP in cytology and cytopathology and the existing gaps in research and implementation that must be addressed for effective application.

The integration of chatbots and NLP in cytology and cytopathology presents transformative opportunities with significant implications for diagnostic and clinical practices [21,22,23,24,25,26,27,28,29,30,31]. These technologies enhance diagnostic accuracy by automating the extraction and classification of information from medical reports, thereby reducing human error and improving precision in data interpretation. Chatbots also revolutionize patient engagement by making information more accessible and supporting complex decision-making, leading to better patient understanding and involvement in healthcare [22,28]. Additionally, NLP tools efficiently analyze unstructured data from medical records, facilitating earlier disease detection and more effective monitoring [24,29]. Moreover, these technologies streamline clinical workflows by automating routine tasks like data entry and report generation, allowing healthcare professionals to focus more on patient care and increasing operational efficiency [23,25]. The integration of chatbots and NLP with electronic health records enables personalized care through tailored recommendations and follow-ups based on individual patient data [31]. In summary, chatbots and NLP have the potential to revolutionize traditional diagnostic approaches in cytology and cytopathology by introducing advanced automated analyses and enhancing cellular abnormality detection. Addressing the challenges associated with these technologies could significantly improve both diagnostic accuracy and patient care.

Table 3 outlines the opportunities and key references.

The overview emphasizes several key areas requiring further investigation to maximize the potential of chatbots and NLP in cytology and cytopathology. Addressing these areas is crucial for advancing these technologies and overcoming their inherent challenges.

*Data standardization and integration*

Suggestion: Develop and test frameworks for standardizing data across different systems to ensure compatibility and seamless integration with chatbots and NLP tools. This is essential for achieving consistency and interoperability in clinical applications.

Challenges: Variability in data formats and integration issues can hinder the adoption and effectiveness of these technologies. Establishing universally accepted standards and protocols is vital to overcome these obstacles [21,22,23].

*Bias and fairness in AI systems*

Suggestion: Investigate methods to detect and mitigate biases in AI algorithms, ensuring equitable outcomes across diverse populations by training models on diverse datasets and implementing continuous performance monitoring.

Challenges: Addressing biases requires dealing with varied datasets and maintaining vigilance against discriminatory practices. Rigorous testing and validation across different demographic groups are necessary to mitigate these risks [21,23,24].

*Clinical validation and accuracy*

Suggestion: Conduct large-scale, longitudinal studies to validate the accuracy and reliability of chatbots and NLP tools in real-world clinical settings. This research is needed to confirm the effectiveness of these tools compared to traditional diagnostic methods.

Challenges: Ensuring accuracy in various clinical contexts and overcoming skepticism from traditional diagnostic practices pose significant challenges. Comprehensive validation efforts are essential to gain acceptance and trust [25,27,29].

*Patient engagement and education*

Suggestion: Explore innovative designs for user-friendly chatbots that enhance patient education and engagement in healthcare processes. Effective interactions through chatbots could lead to improved health outcomes and increased patient involvement.

Challenges: Designing intuitive and accessible chatbot interfaces while integrating them seamlessly into existing patient care workflows are critical hurdles. Addressing these challenges will be key to enhancing patient engagement [26,28].

*Ethical and legal considerations*

Suggestion: Develop comprehensive guidelines and frameworks to address ethical concerns, data privacy, and legal implications of using AI in healthcare. Establishing clear regulations and ethical standards is essential for the responsible deployment of AI technologies.

Challenges: Navigating complex regulatory landscapes and ensuring compliance with privacy laws and ethical standards present significant challenges. Effective frameworks are necessary to protect patient information and ensure responsible use of AI technologies [30,31].

Table 4 reports a sketch of the areas needing broader investigation and their associated challenges.

## 4. Discussion

### 4.1. Added Value of the Review

The narrative review indicates that research into the application of chatbots and natural language processing (NLP) in cytology and cytopathology is still in its early stages. A thorough search of PubMed identified only 15 relevant studies, which comprise 1 comment, 2 editorials, 1 letter, and a single review article. This limited output suggests that the integration of these technologies into the fields of cytology and cytopathology is just beginning to gain traction. The presence of only one review article highlights a nascent academic interest, reflecting early exploration rather than established research.

Despite the modest body of literature, there is an emerging focus on the potential of chatbots and NLP in these disciplines. This initial research indicates foundational work and a budding interest that lays the groundwork for more extensive investigations. As the field matures, addressing existing research gaps will be crucial to unlocking the full potential of these technologies in clinical applications.

The review contributes by detailing how chatbots and NLP could enhance diagnostic accuracy through automated extraction and classification of information from medical reports, thereby minimizing human error and improving precision. Additionally, it discusses the potential for these technologies to streamline clinical workflows, allowing healthcare professionals to allocate more time to patient care instead of administrative tasks.

Moreover, the review highlights the role of chatbots in enhancing patient engagement by providing accessible information and facilitating informed decision-making, which fosters better understanding and involvement in healthcare.

It also addresses significant challenges, such as the need for data standardization, seamless integration with existing healthcare systems, and the mitigation of biases in AI. Overcoming these challenges is essential for realizing the full potential of these technologies in clinical practice.

**Figure 1:**
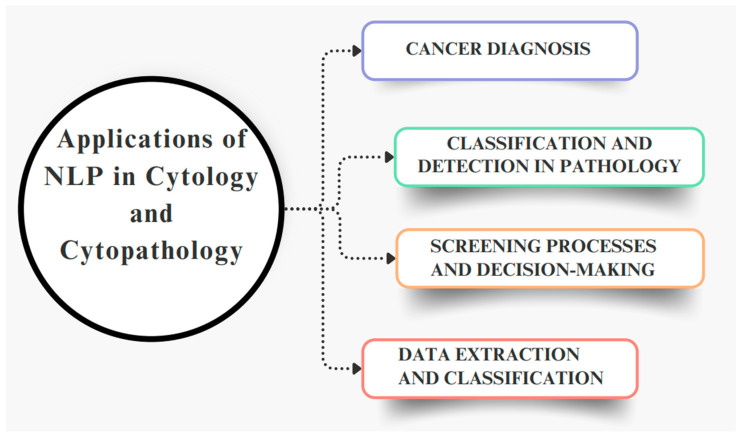
The individuated areas of application of NLP in cytology and cytopathology.

Overall, this review offers a forward-looking analysis of how chatbots and NLP could transform medical diagnostics and patient care in cytology and cytopathology, while also pinpointing critical challenges and opportunities for future development. Figure 1 presents a summary of the applications of NLP in cytology and cytopathology, derived from the analysis of the included articles. The use of NLP is categorized into four macro areas based on the converging themes addressed in the articles. This figure provides an immediate overview of the analyzed roles of NLP as described in the literature.

### 4.2. A Comparison with the State-of-the-Art in Radiology and Histology

#### 4.2.1. Analysis

It is intriguing to consider the findings of this overview in relation to other imaging domains, particularly digital pathology, which shares a domain with cytology and cytopathology. Comparing these insights to the field of radiology is also relevant, as radiology has seen a faster integration into digital health, largely due to its early adoption of DICOM standards.

The rapid integration in radiology provides valuable lessons for digital pathology. Adopting similar standardization and interoperability measures could accelerate the benefits of advanced technologies like chatbots and NLP within digital pathology. The swift adoption of clear and well-defined protocols in radiology underscores the importance of such standards in facilitating effective technology integration and enhancing clinical workflows. Implementing analogous strategies in digital pathology could drive significant advancements and smoother integration of new technologies into clinical practice.

*In comparison to the scientific output in histology and histopathology* as recorded in PubMed, it is evident that research in these domains, which began earlier in 2002 rather than in 2012 [32,33], is significantly more extensive. Specifically, there are 67 publications in histology and histopathology compared to just 15 in cytology and cytopathology, indicating that the latter constitutes only 22.38% of the total output. This difference in the number of scientific publications likely stems from the greater challenges associated with managing digital cytology and cytopathology. The complexity involved in handling the focus function—critical in this field—requires sophisticated solutions and substantial memory resources [34].

Furthermore, PubMed shows that there are five review articles in histology and histopathology [33], maintaining a proportion consistent with the greater volume of research in these fields. These recent reviews [22,35,36,37,38] on the application of NLP and chatbots in pathology reveal (the contribution in [22] faced both the two fields of cytology and histology) a promising landscape for these technologies, emphasizing their potential to revolutionize diagnostic processes and enhance clinical workflows.

*Key insights and contributions*

*Integration of Large Language Models (LLMs) in digital pathology:*

Cheng J’s review [35] discusses how LLMs can be transformative in pathology by automating the extraction and interpretation of complex medical texts. These models can streamline the process of data management, assisting pathologists in efficiently handling extensive reports and improving diagnostic accuracy. By processing vast amounts of textual data, LLMs can support pathologists in making more informed decisions, thus enhancing overall diagnostic efficiency.

*Role of ChatGPT in cancer diagnosis:*

Malik S and Zaheer S [22] explore the use of ChatGPT in aiding cancer diagnosis. ChatGPT, with its advanced conversational capabilities, can assist pathologists by providing preliminary interpretations and insights based on large datasets. This support can significantly reduce the time required for diagnosis and help in managing complex cases more effectively. The ability of ChatGPT to offer contextually relevant suggestions underscores its potential as a valuable tool in pathological diagnostics.

*NLP in the detection of Barrett’s esophagus:*

Patel A and colleagues [36] highlight the role of AI, including NLP, in the early detection of Barrett’s esophagus. NLP can analyze patient records and endoscopic images to improve early diagnosis and monitoring. This review underscores the utility of NLP in parsing through extensive clinical data, making it easier to identify patterns and abnormalities that might otherwise be overlooked, thereby facilitating timely and accurate diagnoses.

*AI and NLP [37] in managing ulcerative colitis:*

Sinonquel P et al. [37] discuss the application of AI in ulcerative colitis, focusing on how NLP can assist in managing and interpreting clinical data. The review points out that NLP technologies can enhance the analysis of patient information and the research literature, leading to better disease management and treatment planning. By integrating NLP into clinical practice, healthcare providers can gain deeper insights into patient conditions and make more informed treatment decisions.

*AI and hybrid imaging in oncology:*

Sollini M [38] and colleagues examine the synergy between AI, including NLP, and hybrid imaging techniques in oncology. The review illustrates how AI can enhance the interpretation of complex imaging data, supporting personalized medicine approaches. NLP can aid in analyzing and correlating imaging findings with clinical data, offering a more comprehensive view of patient conditions and facilitating tailored treatment strategies.

*A comparison with the field of digital radiology reveals a significantly larger volume of research output,* with Pubmed showing [39] studies dating back to 1993, particularly in chatbot applications. In contrast, the field of cytology and cytopathology has produced only 2.1% of the scientific output seen in radiology, i.e., 15 papers versus 684 papers.

In the radiology sector, a comprehensive search on PubMed indicates the production of 71 review articles, including 14 systematic reviews. This substantial number of reviews highlights a higher level of consolidation and advancement in radiology compared to cytology/cytopathology, which has only one review, and histology and histopathology (with five reviews).

Figure S3 (see Supplementary Materials) show the different paper productions in the three fields cytology/cytopathology (A), histology/histopathology (B), and radiology (C), with the number/percent of reviews (all categories).

Figure S4 (see Supplementary Materials) shows the temporal trend of published articles on the use of NLP in the fields of histology, cytology, and radiology. Despite the significant differences in the number of articles published in these three fields, the figure shows that most articles in all three fields have been published within the last five years. This indicates a remarkable growth period for NLP applications in these different medical fields.

The early and extensive research efforts in radiology suggest a more developed and integrated application of digital technologies, including chatbots, compared to the relatively nascent state of these technologies in cytology and cytopathology. This disparity underscores the need for increased research and development in cytology and cytopathology to achieve similar levels of advancement and integration as seen in radiology. Even considering just the five most recent systematic reviews, interesting prospects emerge [40,41,42,43,44].

The recent systematic reviews provide valuable insights into the role of NLP and ChatGPT within radiology.

*ChatGPT and assistive AI in structured radiology reporting*

Sacoransky E et al. [40] highlight ChatGPT’s emerging role in structured radiology reporting, which is an area traditionally dominated by image analysis. The study demonstrates how ChatGPT can enhance reporting accuracy and streamline workflows by automating and improving the organization of radiological data.

*ChatGPT in radiology: performance and limitations*

Keshavarz P et al. [41] evaluate ChatGPT’s effectiveness across 84.1% of radiology studies, but also addresses significant limitations and potential pitfalls. It underscores the necessity for extensive, multicenter studies to fully assess ChatGPT’s accuracy and reliability in clinical settings

*BERTs in radiology: NLP applications*

Gorenstein L [42] explores the transformative impact of bidirectional encoder representations from transformers (BERTs) on radiology. A BERT’s bidirectional context understanding enhances NLP applications, which could lead to more sophisticated analysis and interpretation of radiological texts and reports.

*Current applications and future potential of ChatGPT*

Temperley HC et al. [43] note that while ChatGPT shows potential, it currently exhibits data inaccuracies, especially regarding interventional radiology procedures. Despite these challenges, it highlights ChatGPT’s strategic potential for roles such as tele-mentoring and providing second opinions, particularly when integrated with radiological imaging.

*AI tools in medicine: systematic review and meta-analysis*

Younis HA et al. [44] in a comprehensive review outlines ChatGPT’s adaptability and its promise for transforming medical practices. It emphasizes ChatGPT’s potential to enhance patient care, streamline interactions among healthcare professionals, and support innovative applications such as tele-mentoring.

#### 4.2.2. Emerging Recommendations from Radiology and Histology

The findings from the integration of technologies in radiology and digital pathology offer several valuable lessons for the field of cytology.

Standardization and interoperability: The successful adoption of DICOM standards [34] in radiology [40,41,42,43,44] emphasizes the necessity for clear and well-defined protocols in cytology. Implementing similar standardization and interoperability measures could streamline data management and enhance clinical workflows in cytology. Such frameworks would facilitate effective integration of advanced technologies, including Natural Language Processing (NLP) and chatbots, which have shown promise in enhancing diagnostic accuracy.

Increased research and development: The significant disparity in scientific output between radiology and cytology highlights the urgent need for enhanced research efforts in the latter. With only 15 publications in cytology compared to 684 in radiology, there is a clear indication that cytology must catch up to harness the benefits of digital technologies [39]. Targeted funding and support for research initiatives in cytology could foster innovation and application of digital tools.

Integration of advanced language models: The potential of Large Language Models (LLMs) in pathology demonstrates their transformative capacity to automate the extraction and interpretation of complex medical texts. In cytology, leveraging LLMs could aid pathologists in efficiently managing extensive reports and improve overall diagnostic accuracy. This can lead to better-informed decision-making and streamlined workflows.

Adoption of AI tools: The application of AI, including NLP, in diagnosing conditions like Barrett’s esophagus and managing diseases such as ulcerative colitis illustrates the capabilities of these technologies in parsing vast clinical data [36,37]. For cytology, adopting similar AI-driven approaches could facilitate timely and accurate diagnoses by analyzing complex datasets that are often challenging to interpret manually.

Collaboration and cross-disciplinary learning: The experiences in radiology [40,41,42,43,44] underscore the importance of interdisciplinary collaboration. Encouraging partnerships between cytologists and radiologists could promote knowledge exchange and accelerate the integration of technologies in cytology. Such collaboration may also lead to the development of innovative solutions tailored to the unique challenges faced in cytology.

Focus on systematic reviews: The higher number of systematic reviews in radiology (71 compared to only 1 in cytology) indicates a greater level of consolidation in the field [39]. Implementing a systematic review process in cytology could enhance the rigor and visibility of research findings, fostering a more evidence-based approach in clinical practice.

Education and training: Continuous education on emerging technologies is vital for cytology professionals. Investing in training programs focused on NLP and AI tools can empower practitioners to effectively integrate these technologies into their diagnostic processes, like in histology [22,35,36,37,38] and radiology [40,41,42,43,44]. Such initiatives would ensure that cytologists are equipped to leverage advancements in technology to improve patient care.

In summary, the integration of lessons learned from radiology and digital pathology can significantly advance the field of cytology. By prioritizing standardization, investing in research, adopting advanced technologies, and fostering collaboration, cytology can enhance its diagnostic capabilities and ultimately improve patient outcomes.

### 4.3. Limitations

The proposed study, described as a narrative review, has inherent limitations due to the methodology and inclusion/exclusion criteria applied. The absence of conference studies results in a potential lack of updates on ongoing research and recent advances, excluding preliminary data that may not have been officially published yet. Additionally, the exclusion of local studies and/or guidelines in local languages restricts the analysis to contributions published in English and international guidelines, potentially overlooking specific and relevant insights from particular cultural or clinical contexts. This limits the understanding of regional variations in clinical practices and treatment protocols.

## 5. Conclusions

This narrative review emphasizes the emerging role of chatbots and Natural Language Processing (NLP) in cytology and cytopathology, highlighting that this area is still in its formative stages. With only 11 relevant studies identified, there is an initial but growing interest in these technologies.

The review identifies significant opportunities for chatbots and NLP to enhance diagnostic accuracy by automating information extraction and classification from medical reports. This automation can reduce human error and improve precision. Moreover, chatbots have the potential to transform patient engagement by making medical information more accessible, thus supporting decision-making and enhancing patient involvement in healthcare. Additionally, NLP can streamline clinical workflows by automating routine tasks, allowing healthcare professionals to prioritize patient care. Integrating these technologies with electronic health records could also lead to more personalized patient care through tailored recommendations.

However, several challenges remain. Key issues include the need for data standardization and integration, addressing biases in AI algorithms, and validating these technologies through large-scale studies to confirm their accuracy in clinical settings. Other challenges encompass designing user-friendly chatbots to improve patient engagement and establishing comprehensive guidelines to address ethical, privacy, and legal concerns surrounding AI deployment in healthcare.

Compared to fields like radiology, which have seen faster integration of digital technologies, cytology and cytopathology must accelerate their efforts. The lessons learned from radiology’s early adoption of standardized protocols and extensive research can inform the integration of chatbots and NLP into digital pathology. The more advanced research in histology underscores the need for increased efforts in cytology and cytopathology.

In summary, while the integration of chatbots and NLP into cytology and cytopathology holds great promise for transforming diagnostic practices and patient care, addressing the identified challenges is crucial for realizing their full potential and advancing the field.

## Figures and Tables

**Table 1:** Focus areas and categorization in cytopathology: the role of chatbots and NLP in advancing diagnostic precision.

Study	Focus	Category	Specific Area
Mese et al. [21]	Utilizes ChatGPT to assist in developing a deep learning model for thyroid nodule analysis.	Deep learning model development and AI-assisted medical imaging	Thyroid nodule analysis
Malik and Zaheer [22]	Explores ChatGPT’s role in aiding pathological diagnosis of cancer through improved data processing.	AI integration in pathology and cancer diagnosis	Pathological diagnosis enhancement
Giarnieri and Scardapane [23]	Discusses advanced machine learning and AI applications in cytopathology for classification, detection, and segmentation.	Computational pathology and machine learning in cytology	Cytopathology classification and detection
Rozova et al. [25]	Uses NLP for automated surveillance and classification of invasive fungal infections from histopathology reports.	NLP in automated disease surveillance and classification	Invasive fungal infections
Hsu et al. [26]	Applies NLP techniques for accurate classification of free-text cervical biopsy diagnoses.	NLP for pathology report classification	Cervical biopsy classification
Nandish et al. [27]	Employs NLP for multilevel and multiclass classification of breast lesions based on EHRs.	NLP for multiclass classification in breast pathology	Breast lesion classification
Selmouni et al. [28]	Assesses the impact of a chatbot-based decision aid to enhance participation in cervical cancer screening programs.	Chatbot-based decision support in public health screening	Cervical cancer screening participation
Oliveira et al. [29]	Develops and validates an NLP algorithm for extracting and classifying data related to HPV-associated cancers.	NLP for HPV cancer surveillance and data extraction	HPV-associated cancers surveillance
Wagholikar et al. [30]	Describes a decision tree-based clinical decision support system for cervical cancer screening and surveillance.	Automated decision support systems for cancer screening	Cervical cancer screening recommendations
Nguyen et al. [31]	Develops a system for classifying cancer-notifiable pathology reports using NLP and symbolic reasoning.	NLP for cancer registry notifications and pathology report classification	Cancer-notifiable reports classification

**Table 2:** Broad Areas of Application.

Broad Area	Studies	Focus	Examples
AI and NLP in cancer diagnosis	[21] Mese et al., [22] Malik and Zaheer, [25] Rozova et al., [27] Nandish et al., and [29] Oliveira et al.	Development and application of AI/NLP for improving cancer diagnosis and surveillance.	Thyroid nodule analysis, invasive fungal infections, and breast lesion classification
NLP for pathology and cytopathology	[23] Giarnieri and Scardapane, [25] Rozova et al., [26] Hsu et al., and [27] Nandish et al.	Utilizing NLP and machine learning for classification and detection in pathology and cytology.	Cytopathology classification and histopathology report analysis
AI-enhanced screening and decision support	[28] Selmouni et al., [30] Wagholikar et al., and [31] Nguyen et al.	Integration of AI to improve screening processes and decision-making in public health.	Cervical cancer screening participation and cancer registry notifications
Data extraction and classification with NLP	[26] Hsu et al., [27] Nandish et al., [29] Oliveira et al., and [31] Nguyen et al.	Use of NLP for extracting and classifying clinical data from unstructured text.	Cervical biopsy classification, HPV-associated cancers surveillance, and cancer-notifiable reports classification

**Table 3:** Opportunities and key references.

Opportunity	Description	Key References
Enhanced diagnostic accuracy	Automates the extraction and classification of medical information, reducing human error and improving diagnostic precision.	[21,27]
Streamlined clinical workflows	Automates routine tasks like data entry and report generation, boosting workflow efficiency and allowing more focus on patient care.	[23,25]
Improved patient engagement	Facilitates accessible information and supports decision-making, enhancing patient understanding and involvement.	[22,28]
Efficient data management	Analyzes unstructured data for better disease detection and monitoring, enabling earlier interventions.	[24,29]
Early detection and monitoring	Leverages advanced AI algorithms for early disease detection and continuous monitoring, leading to timely interventions.	[26,30]
Personalized patient care	Integrates with electronic health records to provide tailored recommendations and follow-ups, offering personalized patient care.	[31]

**Table 4:** Areas needing broader investigation and their associated challenges.

Area.	Suggestion for Broader Investigation	Challenges	References
Data standardization and integration	Develop and test frameworks for standardizing data across different systems to ensure compatibility and seamless integration with chatbots and NLP tools.	Variability in data formats and integration issues can hinder the adoption and effectiveness of technology.	[21,22,23]
Bias and fairness in AI systems	Investigate methods to detect and reduce biases in AI algorithms and ensure equitable outcomes across diverse populations.	Addressing biases requires diverse datasets and continuous monitoring to prevent discriminatory practices.	[21,23,24]
Clinical validation and accuracy	Perform large-scale, longitudinal studies to validate the accuracy and reliability of chatbots and NLP tools in real-world clinical settings.	Ensuring accuracy in varied clinical contexts and overcoming skepticism from traditional diagnostic practices.	[25,27,29]
Patient engagement and education	Explore innovative approaches to design user-friendly chatbots that effectively enhance patient education and engagement in their healthcare processes.	Creating intuitive and accessible chatbot interfaces while integrating them into existing patient care workflows.	[26,28]
Ethical and legal considerations	Develop comprehensive guidelines and frameworks addressing ethical concerns, data privacy, and the legal implications of using AI in healthcare.	Navigating regulatory landscapes and ensuring compliance with privacy laws and ethical standards.	[30,31]

## Supplementary Materials

The following supporting information can be downloaded at: https://www.mdpi.com/article/10.3390/bioengineering11111134/s1, Figure S1: Temporal trend of studies focusing on the use of NLP in citology or citopathology; Figure S2: Comparison of temporal trends in articles published using two different search keys; Figure S3: Scientific production in the three fields of cytology/cytopathology (A) histology/histopathology (B), and radiology (C). In reviews are comprised all the review categories; Figure S4: Temporal trend of articles published on the use of chatbot and NLP in histology/histopathology and cytology/cytopathology and radiology; Section S1: The narrative checklist; Table S1: ANDJ checklist; Section S2: Analytical Summary: Advances in Chatbot/NLP Applications in Medical Diagnostics.
