# Supplementary material for: Revolutionizing Cytology and Cytopathology with Natural Language Processing and Chatbot Technologies: A Narrative Review on Current Trends and Future Directions
Source: Bioengineering (Basel). 2024 Nov 11;11(11):1134. doi: 10.3390/bioengineering11111134 (PMC11592174; doi:10.3390/bioengineering11111134)
Supplement: Supplementary file 1 [file bioengineering-11-01134-s001.zip › bioengineering-3180764-supplementary.pdf]

bioengineering

Review

Revolutionizing Cytology and Cytopathology with Natural Language Processing and Chatbot Technologies: A Narrative Review on Current Trends and Future Directions

Andrea Lastrucci [1,†], Enrico Giarnieri [2,†], Elisabetta Carico [2] and Daniele Giansanti [3,*]

[1] Department of Allied Health Professions, Azienda Ospedaliero-Universitaria Careggi, 50134 Florence, Italy; andrea.lastrucci@unifi.it
[2] Facoltà di Medicina e Psicologia, Sede Ospedale S. Andrea via di Grottarossa 1035, Università Sapienza, 00189 Roma, Italy; enrico.giarnieri@uniroma1.it (E.G.); elisabetta.carico@uniroma1.it (E.C.)
[3] Centro TISP, Istituto Superiore di Sanità, via Regina Elena 299, 00161 Rome, Italy
* Correspondence: daniele.giansanti@iss.it
† These authors contributed equally to this work.

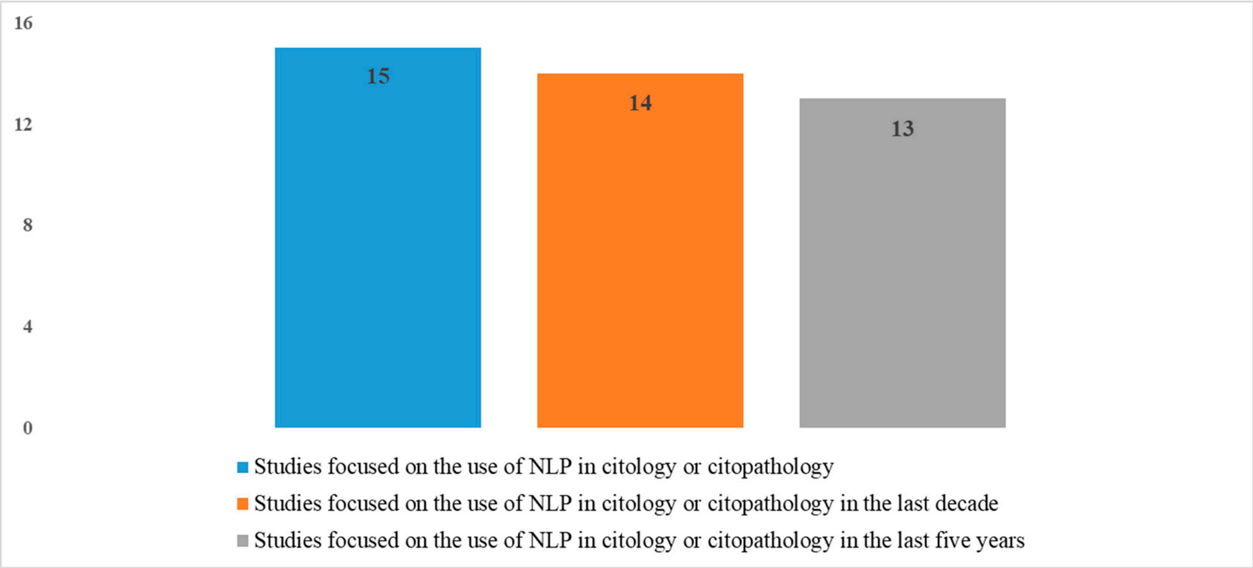

Figure S1. Temporal trend of studies focusing on the use of NLP in citology or citopathology.

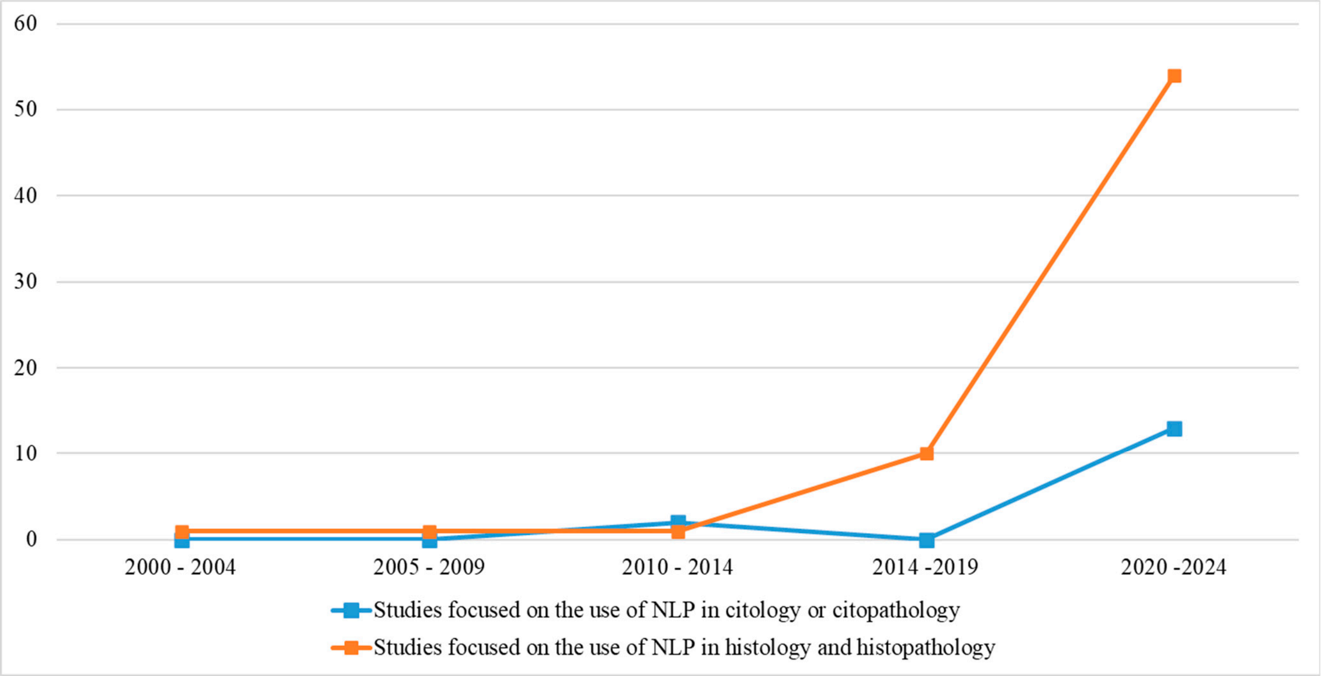

Figure S2. Comparison of temporal trends in articles published using two different search keys.

.

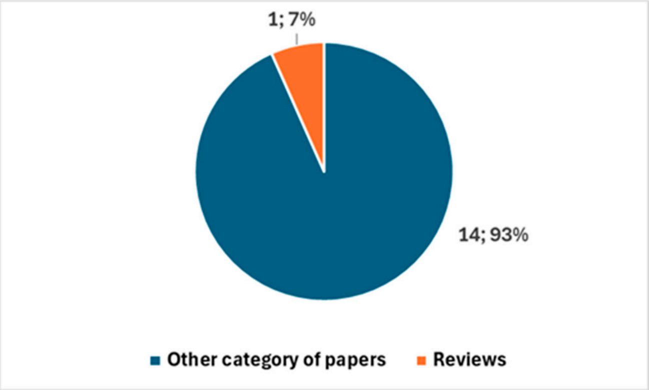

(A).

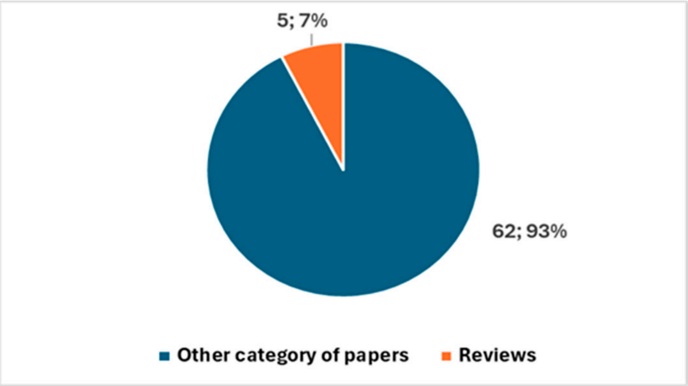

(B).

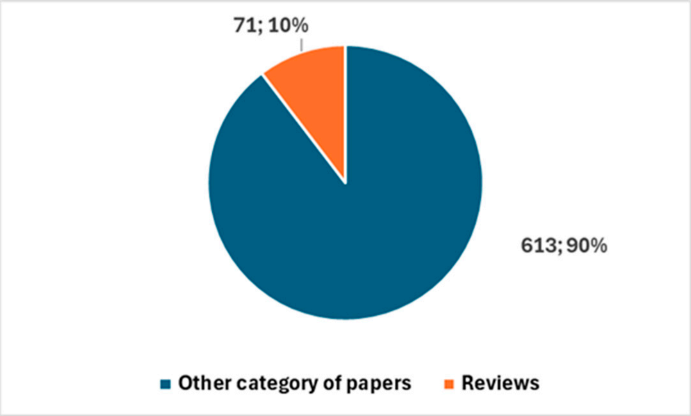

(C).

Figure S3. Scientific production in the three fields of cytology/cytopathology (A) histology/histopathology (B), and radiology (C). In reviews are comprised all the review categories.

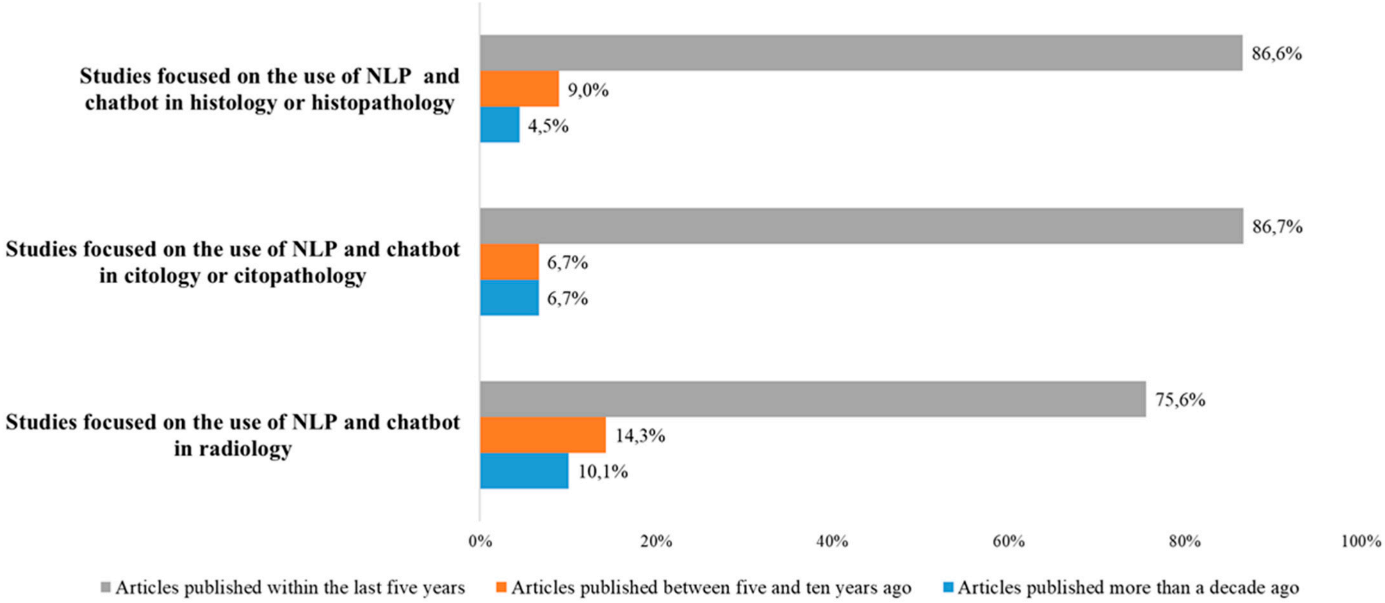

Figure s. 4. Temporal trend of articles published on the use of chatbot and NLP in histology/histopathology and cytology/cytopathology and radiology.

Section S1 The narrative checklist

The **ANDJ checklist** consists of critical sections that guide authors in crafting comprehensive narrative reviews.

Title

Checklist Item 1: The title must explicitly identify the report as a **narrative review**. This clarity informs readers about the nature of the article from the outset, setting the stage for the review's content.

Abstract

Checklist Item 2: The abstract should deliver an **unstructured summary** that includes the background, objective, a brief summary of the review, and implications for future research, clinical practice, or policy development. This succinct overview is vital for quickly conveying the significance and findings of the review to the audience.

Introduction

Checklist Item 3: In the introduction, authors must describe the **rationale** for the review in the context of existing knowledge. This section highlights the importance of the topic and establishes the need for the review.

Checklist Item 4: Authors should specify the **key questions** identified for the review topic, providing a clear focus for the narrative.

Methods

Checklist Item 5: The methods section should detail the **research selection process**, including the years considered, language, publication status, study design, and databases used. This transparency ensures that readers understand how the literature was identified and selected.

Discussion/Summary

Checklist Item 6: The narrative discussion must address key aspects: it should discuss the research reviewed, highlighting fundamental findings, limitations, and quality of the studies examined, and emphasize the need for future research.

Checklist Item 7: Finally, the summary should provide an overall interpretation of the narrative review, contextualizing it for health professionals, policy development, and future research directions.

Table S1. ANDJ checklist

Section/topic	#	Checklist item	Reported on page
TITLE			
	1	Identify the report as a Narrative Review of …	Page 1
Abstract			
	2	Provide an unstructured summary including, as applicable: background, objective, brief summary of narrative review and implications for future research, and clinical practice or policy development.	Page 1
INTRODUCTION			
Rationale/background 3	3	Describe the rationale for the review in the context of what is already known.	Page 4
Objectived	4	Specify the key question(s) identified for the review topic	Page 4
METHODS			
Research selection	5	Specify the process for identifying the literature search (eg, years considered, language, publication status, study design, and databases of coverage).	Page 4-5 and 16-17
DISCUSSION/SUMMARY			
Narrative	6	Discuss: 1) research reviewed including fundamental or key findings, 2) limitations and/or quality of research reviewed, and 3) need for future research.	Page 5-13
Summary	7	Provide an overall interpretation of the narrative review in the context of clinical	Page 13-16

		practice for health professionals, policy development and implementation, or future research	

Section S2 Analytical Summary: Advances in Chatbot/NLP Applications in Medical Diagnostics

The overview has been complemented by means of anallitical summaries with focus on the contribution of the chatbot/NLP.

1. Integration of ChatGPT in Medical Imaging Analysis

The study by Mese et al. [21] illustrates the use of ChatGPT to enhance a deep learning model for thyroid nodule analysis using ultrasound images. ChatGPT contributed to various stages of model development, including code writing, preprocessing, and optimization. The resulting model achieved an accuracy of 0.81, with high precision and recall rates in detecting both benign and malignant thyroid nodules. This application demonstrates how AI, particularly ChatGPT, can support the creation of robust diagnostic tools, potentially improving diagnostic accuracy and efficiency in medical imaging.

2. Role of ChatGPT in Pathological Diagnosis

Malik and Zaheer [22] discuss the integration of ChatGPT in cancer pathology, highlighting its role in processing complex data from cytopathology and histopathology. The use of ChatGPT can streamline pathology workflows by aiding in the interpretation of digital slides and implementing advanced diagnostic algorithms. Despite its potential, challenges such as digital slide integration, bias, and legal concerns remain. Addressing these issues is crucial for leveraging ChatGPT effectively in pathology.

3. Machine Learning and Deep Learning in Cytopathology

Giarnieri and Scardapane [23] explore advancements in machine learning and deep learning for cytopathology, emphasizing their role in classification, detection, and segmentation of pathological data. These technologies have shown impressive accuracy in analyzing large image datasets, moving beyond traditional microscopy. The integration of AI with technologies like augmented reality, virtual reality and other advanced technologies, including the NLP is poised to further transform cytopathology, although challenges such as dataset availability and training requirements persist.

4. Natural Language Processing for HPV Surveillance

Uusküla et al. [24] employed text mining and NLP to study HPV prevalence and cervical cytology in Estonia. By analyzing electronic health records, the study revealed high-risk HPV types and assessed the impact of vaccination on cytological findings. NLP techniques proved effective in extracting and analyzing relevant data from health records, highlighting the potential for NLP in improving surveillance and prevention strategies.

5. NLP for Invasive Fungal Infection Surveillance

Rozova et al. [25] focused on using NLP for detecting invasive fungal infections (IFIs) from cytology and histopathology reports. By incorporating concept-level annotations, their model achieved a high performance in identifying IFI-positive reports, with an improved classifier performance compared to traditional methods. This approach illustrates NLP's potential in automating and enhancing the accuracy of infection surveillance in clinical settings.

6. NLP in Cervical Biopsy Diagnosis

Hsu et al. [26] utilized NLP to classify cervical biopsy free-text diagnoses. Their use of FastText™ for classification demonstrated high accuracy and concordance with manual annotations. This study underscores NLP's capability to handle large volumes of unstructured medical text, thereby supporting efficient and accurate pathology diagnosis.

7. Automated Classification of Breast Lesions

Nandish et al. [27] applied NLP to automate the classification of breast lesions from cytopathology reports. By leveraging machine learning techniques, including random forests and neural networks, the study achieved high classification accuracy and robust performance across multiple breast lesion types. This demonstrates NLP's potential to significantly streamline and improve the classification of complex medical data.

8. AI-Assisted Cervical Cancer Screening

Selmouni et al. [28] are developing a chatbot-based decision aid to enhance cervical cancer screening participation among vulnerable women in France. This intervention aims to overcome barriers such as lack of information and improve participation rates in screening programs. The study will evaluate the effectiveness of the chatbot in increasing engagement and screening outcomes.

9. NLP for Cervical and Anal Cancer Surveillance

Oliveira et al. [29] developed an NLP algorithm to identify cervical and anal cancers from pathology reports. The algorithm demonstrated high accuracy in detecting abnormal cytology and HPV tests, highlighting NLP's utility in automating cancer surveillance and improving diagnostic efficiency.

10. Automated Recommendation Systems for Cervical Cancer

Wagholikar et al. [30] created a decision tree-based clinical decision support (CDS) system for cervical cancer screening. This system integrates national guidelines to provide tailored recommendations for abnormal findings, improving adherence to best practices and overall diagnostic accuracy.

11. Automatic Classification for Cancer Registry Notifications

Nguyen et al. [31] developed a system for classifying pathology reports as cancer-notifiable using NLP. The system demonstrated high sensitivity and specificity, showcasing how NLP can facilitate efficient cancer registry notifications and enhance the reliability of cancer surveillance systems.

The analytical summary highlights that the integration of chatbot and NLP technologies in cytology/cytopathology medical diagnostics represents a significant advancement in the field. From improving diagnostic accuracy in medical imaging and pathology to enhancing surveillance and screening programs, these AI-driven tools offer promising solutions to various challenges in healthcare. Despite their potential, addressing issues related to data integration, algorithm bias, and ethical considerations remains essential for their successful implementation and widespread adoption.
